# Supplementary material for: Predicting visual function by interpreting a neuronal wiring diagram
Source: Nature. 2024 Oct 2;634(8032):113–23. doi: 10.1038/s41586-024-07953-5 (PMC11446822; doi:10.1038/s41586-024-07953-5)
Supplement: Supplementary file 6 — CRF and ERF predictions for individual TmY4 and TmY9 cells. Analogous to Supplementary Data 3, but for TmY target types. Shown are the top four monosynaptic pathways, the strongest pathway passing through each of the top ten intermediary types (ranking from Extended Data Fig. 7), and the trisynaptic pathway Tm1–TmY–Dm3–TmY (see the section entitled Prediction of spatial normalization). [file 41586_2024_7953_MOESM6_ESM.zip › DataS4/TmY9qΓèÑ/720575940641380853.pdf]

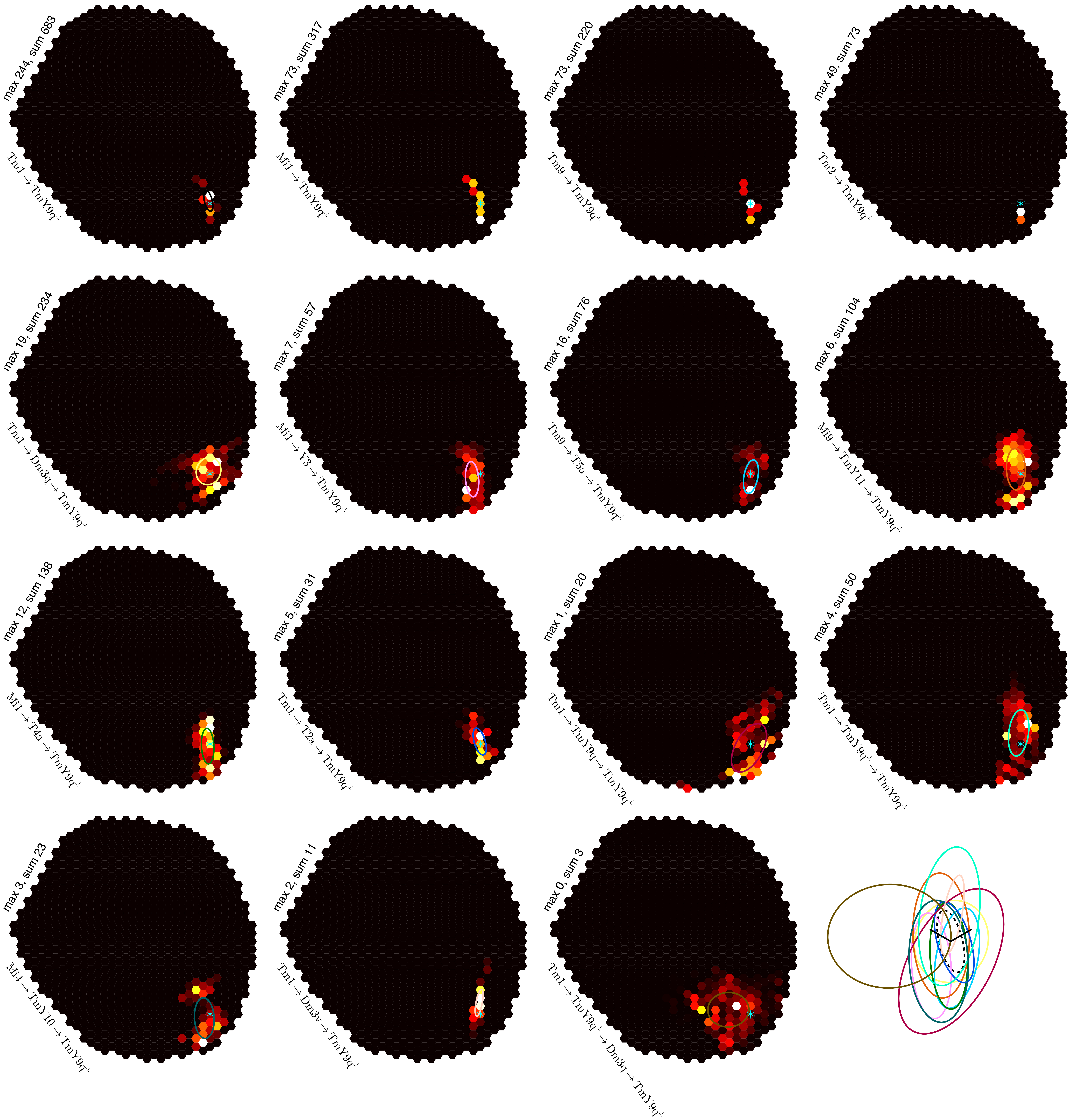

max 244, sum 683
Tm1 → TmY9q⊥
max 73, sum 317
Mi1 → TmY9q⊥
max 73, sum 220
Tm9 → TmY9q⊥
max 49, sum 73
Tm2 → TmY9q⊥
max 19, sum 234
Tm1 → Dm3q → TmY9q⊥
max 7, sum 57
Mi1 → Y3 → TmY9q⊥
max 16, sum 76
Tm9 → T5a → TmY9q⊥
max 6, sum 104
Mi9 → TmY11 → TmY9q⊥
max 12, sum 138
Mi1 → T4a → TmY9q⊥
max 5, sum 31
Tm1 → T2a → TmY9q⊥
max 1, sum 20
Tm1 → TmY9q → TmY9q⊥
max 4, sum 50
Tm1 → TmY9q⊥ → TmY9q⊥
max 3, sum 23
Mi4 → TmY10 → TmY9q⊥
max 2, sum 11
Tm1 → Dm3v → TmY9q⊥
max 0, sum 3
Tm1 → TmY9q⊥ → Dm3q → TmY9q⊥
